# Close female friendships and knowledge of recommended abortion methods in Nigeria and the Democratic Republic of the Congo among a representative sample of reproductive-aged women

**DOI:** 10.3389/frph.2024.1453717

**Published:** 2024-10-31

**Authors:** Selena P. Anjur-Dietrich, Alice Rhoades, Pierre Z. Akilimali, Funmilola M. OlaOlorun, Elizabeth Omoluabi, Suzanne O. Bell

**Affiliations:** ^1^ Department of Population, Family, and Reproductive Health, Johns Hopkins Bloomberg School of Public Health, Baltimore, MD, United States; ^2^ Public Health, Kinshasa School of Public Health, Kinshasa, Democratic Republic of the Congo; ^3^ Department of Community Medicine, College of Medicine, University of Ibadan, Ibadan, Nigeria; ^4^ Department of Statistics and Population Studies, University of the Western Cape, Bellville, South Africa

**Keywords:** abortion, Nigeria, Democratic Republic of the Congo (DRC), social networks, female friendship, abortion knowledge, medication abortion

## Abstract

**Introduction:**

There is a high incidence of unsafe abortion among women in Nigeria and the DRC. Low knowledge of recommended abortion methods [i.e., surgical and medication abortion (MA) pills] is a barrier for women accessing safe abortions. Women often rely on friends for information about abortion methods. Understanding characteristics of women with knowledge of recommended abortion methods, and MA specifically, and how it is influenced by close female friendships may help identify women most at risk of relying on unsafe abortion.

**Methods:**

We used survey data from Performance Monitoring for Action from 11,106 women of reproductive age in Nigeria (April–May 2018) and 3,697 women in Kinshasa and Kongo Central, DRC, (December 2021–April 2022) to produce representative estimates of knowledge of abortion methods at the national and province levels, respectively. We performed bivariate and multivariate logistic regression to determine which characteristics were independently associated with knowing a recommended abortion method, with knowing of MA pills specifically, and to assess our hypothesis that having at least one female confidante would increase one's odds of knowing about these methods.

**Results:**

A minority (26.9%) of women in Nigeria and the majority in Kinshasa (76.7%) and Kongo Central (58.1%) reported having knowledge of at least one recommended abortion method, while knowledge of MA pills was low in all sites. Having at least one close female confidante was associated with increased odds of knowing a recommend abortion method in Nigeria (aOR = 1.50, 95% CI 1.25–1.79) and in Kongo Central (aOR = 2.66, 95% CI 1.40–5.40), and with increased odds of knowing about MA specifically in Kinshasa (aOR = 1.44, 95% CI 1.08–1.93) and Kongo Central (aOR = 3.61, 95% CI 1.28–10.22), but not Nigeria.

**Discussion:**

In legally restrictive contexts where knowledge of recommended abortion methods (particularly medication abortion) is low, having close female friends is related to increased knowledge of recommended abortion methods.

## Background

Induced abortion is legally restricted in most African countries, yet evidence suggests it is a common reproductive health event. Nearly 7 million abortions occur across the continent every year, with 3 in 4 occurring under unsafe conditions, involving a non-recommended method and/or an untrained provider (1). Abortions using recommended methods (including medication abortions and surgical/procedural abortions provided by trained providers) under appropriate conditions are very low-risk medical care; however, unsafe abortions pose a significantly greater risk of complications. Safe abortion results in only 0.7 deaths per 100,000 abortions (2) whereas there are 185 deaths per 100,000 unsafe abortions in sub-Saharan African countries (3). Approximately 10% of maternal deaths in the sub-Saharan African region are due to unsafe abortion (4), and model-based estimates indicate that Western and Central Africa have the highest overall abortion case fatality rate in the world, exceeding 400 deaths per 100,000 abortions (1).

Two countries in this region in which abortion—and specifically unsafe abortion—is common despite legal restrictions are Nigeria and the Democratic Republic of the Congo (DRC). While abortion is only legal to save a woman's life in most Nigerian states, a national study estimated Nigeria's one-year abortion incidence to be 46 per 1,000 women aged 15 to 49 in 2017 (5). Estimates suggest that approximately two-thirds of abortions in Nigeria are highly unsafe, involving a non-clinical provider and methods not recommended by the World Health Organization [i.e., methods other than surgery or medication abortion (MA) pills] (5). In the DRC, abortion was legalized up to 14 weeks’ gestation in a broad range of cases in 2018 through the government's endorsement of the Maputo Protocol (6). No country-level estimates exist, but abortion incidence in Kinshasa, the capital and largest city, is estimated to be 105.3 per 1,000 women of reproductive age and 44.3 in Kongo Central province (7). A recent study estimated that 16.8% of abortions in Kinshasa and 29.9% in Kongo Central involved non-recommended methods obtained from non-clinical sources (e.g., pharmacies, friends, traditional healers) (7).

These unsafe abortions needlessly put women at risk of morbidity and mortality. In Nigeria, there are 512 maternal deaths per 100,000 live births (8), and in the DRC, the most recent study estimates 693 deaths per 100,000 live births (significantly higher than the regional average of 546) (9). The morbidity and mortality associated with unsafe abortions in low-resource settings are disproportionately experienced by disadvantaged women (10). In Nigeria, Bell et al. (5) found that women experiencing the most unsafe abortions were significantly more likely to be adolescents, of lower wealth, and have attended no formal schooling, and in the DRC, those with no education similarly experienced the greatest burden of unsafe abortion (7). Prolonged armed conflict in some regions in the DRC has also heightened the prevalence of sexual violence and resulting pregnancies, and studies have documented the particular difficulties faced by survivors in parenting or accessing abortion care (11–13).

Globally, most safe abortions are procedural abortions (also referred to as surgical abortions). However, recent increases in the availability of MA pills (misoprostol with or without mifepristone) presents an opportunity for women in legally restrictive settings to more safely terminate without requiring contact with a medical facility (14). Evidence from multiple contexts suggests that self-managed MA is associated with decreased abortion-related morbidity and severity of complications compared to the negative sequelae associated with use of unsafe termination methods (15, 16). Given that the majority of women in Nigeria and in the DRC rely on non-recommended, unsafe methods to terminate unwanted pregnancies, improving knowledge of and access to MA pills is critical (5, 17). Use of safe abortion and post-abortion care generally varies by social characteristics (5, 18–20). In the DRC, MA use is more common among poor women, including self-managed use of misoprostol without provider consultation (6). In the absence of further legal reform in Nigeria, and while the full implementation of the Maputo Protocol evolves in the DRC, improving the safety of abortions will require expansion of harm reduction models that increase awareness and correct utilization of MA pills for self-management (21, 22).

Understanding women's knowledge of safe abortion methods is an essential first step in efforts that seek to reduce their use of non-recommended methods, as women frequently cite a lack of such knowledge as a barrier to accessing safe abortions (23, 24). However, little is known about the baseline awareness of recommended abortion methods among the general population of Nigerian women and Congolese women. One study using a clinical convenience sample in Nigeria found extremely low (2%–3%) prevalence of knowledge of mifepristone or misoprostol among women seeking abortion (17), though other studies suggest that people with incomplete knowledge about MA can still often have safe and successful abortions (25, 26). An important limitation affecting all of these studies is the use of samples of women seeking abortion, who differ systematically from the general population and likely had already begun gathering relevant information. Further research representing the broader population is needed to identify women who are unaware of recommended abortion methods—and MA pills specifically—and thus most at risk of using an unsafe method, should the need to terminate a pregnancy arise.

One factor that could potentially be important in explaining variation in knowledge of safe abortion methods is one's social network. Close female friendship emerges as an important theme in studies of women's abortion-related decision-making (27, 28), and the extent of sharing one's own abortion experience with others appears to vary by community-level factors including the level of abortion stigma and ease of access to care without breaking anonymity (29). Friends are one of the most common sources of information for women seeking abortion services (15, 27, 30), and women place a high level of trust in the information they receive from friends (31). The role of female friendship has not been explicitly studied in relation to how it may influence abortion-specific knowledge. In some qualitative studies, reliance on female friendship has led women to seek safe abortions (27, 32), while in others, it has led them to use unsafe methods (23, 27, 32, 33). In the DRC, 1 in 3 respondents in a sample of women who terminated pregnancies related to sexual violence reported that friends provided them information about abortion (12). While available evidence suggests there is heterogeneity in the impact that close friends can have on one's abortion trajectory, this research is sparse and nearly all qualitative, limiting further exploration and examination of the independent association of social connectedness on knowledge of safe abortion methods in the general population.

In this study we examine knowledge of safe abortion methods—including procedural and MA pills (misoprostol with or without mifepristone)—among women of reproductive age in Nigeria and two provinces in the DRC. We aim to determine characteristics associated with awareness of safe abortion methods and to evaluate whether having close female friends is associated with knowing at least one safe abortion method and knowing about MA specifically. We hypothesize that women who have one or more close female confidantes will be significantly more likely to know at least one recommended abortion method, and specifically be more likely to know about MA pills given their relative lack of visibility compared to procedural abortion. Findings will address a gap in existing literature and improve our understanding of women's knowledge of safe abortion methods and whether this information is likely to be shared through close female confidantes.

## Methods

### Sampling

This study was based on data from a cross-sectional population-based survey of women of reproductive age (15–49) in Nigeria and two sites in the DRC, Kinshasa and Kongo Central provinces (reflecting urban and rural populations, respectively), conducted by Performance Monitoring for Action (PMA). The Johns Hopkins University Bloomberg School of Public Health oversees PMA and its abortion measurement project and provides technical support. The Centre for Research, Evaluation Resources and Development (CRERD) is the implementing partner in Nigeria, and the Kinshasa School of Public Health is the implementing partner in the DRC.

PMA uses a multi-stage stratified cluster design to collect a nationally and/or sub-nationally representative sample of data from households and women. In Nigeria, seven states were selected, including one state from five of the six zones and two states from the sixth zone (North West zone), where 25% of the total population resides. In the DRC, two provinces were selected: Kinshasa and Kongo Central. Enumeration areas (EAs) or clusters containing approximately 200 households were selected within each state or province using probability proportional to size sampling. Within each EA, 35 households (40 in Lagos state, Nigeria) were randomly selected and all eligible female respondents ages 15–49 were invited to participate in an in-person interview with a female interviewer residing nearby. Interviewers collected verbal informed consent from respondents prior to beginning the interview. This sampling strategy provided a representative sample of reproductive-aged women in Nigeria at the national and state level, and of reproductive-aged women in Kinshasa and Kongo Central separately at the province level. For this study we used data from PMA Nigeria Round 5 (a repeated cross-sectional study), collected between April–May 2018, and from PMA DRC Phase 3 (referring to the third phase of panel data collection), collected in December 2021–April 2022. The final sample included 11,106 women in Nigeria, 2,329 in Kinshasa, and 1,856 in Kongo Central. Ethical approval for this study was obtained from the Institutional Review Board at the Johns Hopkins University Bloomberg School of Public Health, the Comité d'Éthique at the Kinshasa School of Public Health, and the National Health Research Ethics Committee of Nigeria.

### Measures

PMA surveys collect information about women's socioeconomic characteristics, reproductive history, and experiences with family planning methods. The rounds of data collection used in this study included a module on abortion to explore women's knowledge, attitudes, and experiences surrounding abortion. The abortion module began with questions about the experiences of women's close female confidantes. In the survey, investigators defined a confidante as a close female friend or relative between the ages of 15 and 49, residing in the same country, who shares personal information with the respondent and with whom the respondent shares personal information. In Nigeria, respondents were asked how many women fit such a description, while in Kinshasa and Kongo Central, women were asked whether they knew any women who matched this description. For both countries we created a dichotomous exposure variable, classifying each respondent as having no female confidantes or at least one female confidante.^1^

Interviewers then asked about respondent's knowledge of abortion practices in the community where they live. This included a question asking women to list all the methods they had heard of that a woman in her community could use to remove a pregnancy (interviewers noted each method named by selecting corresponding response options in ODK,^2^ and probed respondents to name “anything else?” before moving on). We categorized respondents who reported either procedural abortion or MA pills (misoprostol with or without mifepristone) as having knowledge of recommended abortion methods and those who did not report any known methods or who reported other non-recommended methods (such as other pills, home remedies, or traditional methods) as not having knowledge of recommended abortion methods. This dichotomous knowledge variable was our first outcome variable of interest. We also examined knowledge of MA pills specifically as a dichotomous outcome.

Finally, women were asked about their personal history of abortion with separate questions asking if respondents had previously done something to remove a pregnancy or regulate their period when they were pregnant or worried they were pregnant. A woman was classified as having a history of abortion if she responded yes to either question.

### Analyses

Before beginning analyses, we restricted the analytic data to female respondents who had a complete household and female survey and who were usual residents or slept in the household the night prior to the survey (i.e., the *de facto* population). In the DRC surveys, respondents were reconsented before being asked questions about abortion (including both the confidante question and abortion method knowledge questions) or gender-based violence, and we only include those respondents who completed the abortion module. We conducted bivariate analyses to compare the included and excluded samples. We first conducted univariate analyses to examine the socioeconomic characteristics, reproductive history, knowledge of abortion methods, and number of close female confidantes (0 vs. ≥1) for all women in the analytic population. The specific variables explored included: age, education, marital status, household religion, household ethnicity, wealth quintile, parity, and reported history of abortion. In Nigeria, we further examined urban vs. rural residence and state; because the sampling frame in the DRC (used for the census) does not include urban or rural designations, we do not include this covariate (however, we note that all respondents in Kinshasa live in urban areas while those in Kongo Central reside in a mix of rural and denser areas). We selected these variables *a priori* based on existing literature and our hypotheses regarding factors that would likely confound the relationship between number of confidantes and knowledge of recommended abortion methods. We also report percentages of women with knowledge of individual abortion methods, by study location.

Next, we sought to assess the relationship between these characteristics and the independent and dependent variables. We performed cross-tabulations to determine the characteristics associated with no close female friends to those with at least one. We conducted a similar analysis to compare women with knowledge of a recommended abortion method available in the area where they lived compared to those who did not know a recommended method, and similarly for knowledge of MA pills, specifically. Significance was determined using a design-based F-test to compare the distribution of individual characteristics across the groups using an *alpha* of 0.05.

Finally, we used study site-specific multivariable logistic regressions first to determine which characteristics were independently associated with knowing a recommended abortion method, and second, with knowledge of MA pills. Respondents missing data for any model variables were excluded from these analyses. Our key independent variable was having at least one female confidante. We calculated adjusted odds ratios after adjusting for age (5-year categories), education, marital status, religion, wealth quintile, ethnicity, parity and personal history of abortion using categorical indicator variables, as well as state and residence in Nigeria.^3^ To address the concern of reverse causality, by which personal history of abortion results in increased knowledge of safe abortion methods, we conducted sensitivity tests excluding respondents who reported having had an abortion (tests revealed no impact on our findings and are summarized in Supplementary Figure S1). We also conducted a sensitivity test in which we excluded respondents who knew of both recommended methods, and specifically examined the relationship between having a confidante and knowing of medication but not procedural abortion. Given that the DRC sites differ as Kinshasa is an urban province while Kongo Central is predominantly rural, we also assessed whether stratification by urban/rural residence would reveal further nuance in Nigeria; all models did not differ from the overall findings in Nigeria and so we only report the overall analysis for Nigeria here. We conducted all analyses in Stata IC 15 using survey weights to account for the complex sampling design and clustering, however, we present unweighted sample size numbers.

## Results

### Sample characteristics

In Nigeria, 11,106 women aged 15–49 were in the final analytic sample (response rate 96.8%). The mean age of respondents was 29.1 years (estimate not shown). Most women were married or cohabiting (63.7%) and had one or more children (64.9%; overall sample characteristics shown in the table in Supplementary Table S1). Nearly half of respondents had attended at least some secondary school (46.9%) while 57.1% resided in urban areas. Only 19.0% of the respondents self-reported having done something to terminate a pregnancy or suspected pregnancy. Of the 10,671 who responded to the question about number of close female confidantes and were therefore included in multivariable analyses, 5,883 (54.9%) reported having one or more, while the remaining 4,788 (45.1%) reported no close female confidantes.

The initial samples in the DRC included 2,329 women in Kinshasa (response rate 94.0%) and 1,856 in Kongo Central (response rate 97.8%). The 190 respondents in Kinshasa who did not consent to complete the abortion module of the survey and are therefore excluded from the analytic sample did not differ significantly from the 2,136 who did consent along any background characteristics considered; in Kongo Central, the 295 excluded respondents were more likely to have no education (13.3% vs. 5.4%, *p* = 0.001) and tended to be less wealthy (*p* = 0.04) compared to the 1,561 included respondents.

Respondents in Kinshasa and in Kongo Central were on average 28.2 and 29.4 years old, respectively (overall sample characteristics shown in Supplementary Table S1). Only 41.4% were married in Kinshasa, while 58.9% of women in Kongo Central were married. Most respondents in both settings had at least one child (56.8% in Kinshasa; 76.3% in Kongo Central). Respondents in Kinshasa tended to be more educated than those in Kongo Central (with 94.3% having attended secondary school or higher in Kinshasa, compared to 67.3 in Kongo Central). A similar proportion of respondents in Kongo Central reported ever having had an abortion (18.8%) compared to the Nigerian sample, while this was more common in Kinshasa (27.4%). Most respondents reported having one or more confidantes in both sites (Kinshasa: 68.0%; Kongo Central: 75.9%).

### Characteristics of women by number close female confidantes

We found that women with one or more confidantes differed significantly from those with no confidantes with respect to knowledge of safe methods across all sites, and with regard to some background characteristics in Nigeria and in Kinshasa (Table 1). In Nigeria, a larger portion of women with one or more close confidantes reported knowing of procedural abortion compared to women with no close confidantes (28.4% vs. 8.2%), with no significant difference in MA knowledge. The reverse was true of the DRC samples, where women with confidantes were more likely to know of MA pills compared to those with no confidantes (Kinshasa: 33.3% vs. 25.4%; Kongo Central: 35.3% vs. 13.8%), with no significant difference in procedural abortion knowledge.

**Table 1:** Characteristics of women ages 15–49 in Nigeria, Kinshasa, and Kongo Central by whether they reported having confidantes.

Background characteristics	Nigeria (*N* = 11,106)					Kinshasa (*N* = 2,136)					Kongo Central (*N* = 1,561)				
	No confidantes		Confidantes		*p*-value^b^	No confidantes		Confidantes		*p*-value	No confidantes		Confidantes		*p*-value
	%^a^	*N*	%	*N*		%	*N*	%	*N*		%	*N*	%	*N*	
**Knowledge of medication abortion**					*0*.*069*					**0**.**011**					**0**.**019**
No	91.9	4,426	89.6	5,266		74.6	558	66.7	909		86.2	346	64.7	806	
Yes	8.2	413	10.4	675		25.4	193	33.3	476		13.8	57	35.3	352	
**Knowledge of procedural abortion**					**<0**.**001**					0.291					0.252
No	79.0	3,979	71.6	4,387		30.9	249	26.7	360		59.9	220	50.3	525	
Yes	21.0	860	28.4	1,554		69.2	502	73.3	1,025		40.1	183	49.7	633	
**Age**					**<0**.**001**					0.120					0.383
15–19	17.5	931	19.8	1,215		20.4	141	22.8	323		16.5	73	21.7	275	
20–24	14.9	733	17.5	1,077		20.3	159	21.7	296		15.1	57	17.7	213	
25–29	17.2	837	20.1	1,130		16.2	121	17.1	239		16.1	68	15.6	180	
30–34	15.0	705	15.0	862		10.4	89	12.6	180		13.6	52	13.6	146	
35–39	15.5	679	12.7	742		13.4	96	11.1	152		17.7	68	13.9	160	
40–44	11.7	542	9.2	510		11.3	79	9.6	121		11.1	43	9.5	101	
45–49	8.2	361	5.7	347		8.1	66	5.2	74		10.0	42	8.0	83	
**Education**					**0**.**004**					0.247					0.917
Never	19.0	1,179	14.8	1,000		0.3	4	0.3	5		6.0	28	5.2	53	
Primary	16.5	890	14.2	958		6.2	50	5.0	79		25.6	93	27.9	273	
Secondary	46.9	2,053	48.1	2,743		74.4	554	71.1	997		65.2	265	63.3	776	
Higher	17.6	666	22.8	1,182		19.0	143	23.6	304		3.3	17	3.7	56	
**Marital Status**					**<0**.**001**					**0**.**013**					0.522
Currently married/cohabiting	66.4	3,308	61.1	3,769		47.2	352	38.6	540		60.7	241	58.4	641	
Divorced or separated/widowed	5.5	249	4.3	248		6.6	64	7.3	104		13.2	56	11.4	126	
Never married	28.1	1,229	34.6	1,866		46.2	335	54.1	741		26.1	106	30.3	391	
**Religion (household)**					*0*.*086*					0.212					0.408
Catholic	13.1	563	15.8	961		19.2	143	14.0	213		18.3	80	23.5	266	
Other Christian	44.1	1,648	45.4	2,121		64.0	456	69.6	881		44.1	194	45.1	527	
Muslim	41.2	2,492	36.4	2,588		0.8	5	1.6	19		0.3	2	0.5	7	
Other	1.7	85	2.4	213		16.1	119	14.8	232		37.4	124	30.9	351	
**Wealth**					0.324					**0**.**011**					0.880
Poorest	23.1	1,445	22.2	1,533		18.3	141	18.7	309		12.7	38	15.1	116	
Second poorest	20.3	1,089	20.2	1,363		22.0	175	16.1	239		16.4	51	15.1	144	
Middle	19.5	871	16.4	988		21.3	163	18.8	256		20.1	69	20.2	202	
Second wealthiest	18.1	718	19.4	985		20.8	164	19.3	283		22.8	114	24.8	292	
Wealthiest	19.1	665	21.8	1,014		17.6	108	27.1	298		28.0	131	24.7	404	
**Residence**					*0*.*075*										
Rural	39.3	2,375	44.7	3,077											
Urban	60.7	2,413	55.3	2,806											
**State**					0.403										
Anambra	10.3	484	14.4	869											
Kaduna	10.0	1,139	8.9	1,476											
Kano	14.5	919	11.2	751											
Lagos	22.5	747	21.4	833											
Nasarawa	12.5	604	14.3	861											
Rivers	17.8	542	17.1	673											
Taraba	12.4	353	12.7	420											
**Ethnicity (Nigeria, DRC)**					0.206					0.115					0.793
Hausa Bakongo	22.8	1,725	19.0	1,632		23.8	194	28.3	376		95.0	375	95.1	1,062	
Igbo Bas-Kasai	21.0	806	23.6	1,191		41.8	313	33.2	506		2.3	11	1.5	35	
Yoruba Kasai	13.8	478	13.1	526		13.5	94	16.2	198		1.0	5	1.1	20	
Other	42.4	1,779	44.4	2,533		20.9	150	22.3	305		1.7	11	2.2	40	
**Parity**					**<0**.**001**					**0**.**003**					0.537
0	31.9	1,458	37.9	2,136		38.5	279	45.4	619		21.0	87	24.5	332	
1–2	24.8	1,142	25.8	1,439		25.6	205	28.2	388		27.7	122	29.8	342	
3–4	23.2	1,092	20.6	1,213		19.1	148	16.6	230		22.6	87	20.9	227	
5+	20.1	1,086	15.8	1,087		16.8	119	9.8	147		28.7	107	24.9	256	
**History of abortion**					**<0**.**001**					**0**.**009**					*0*.*095*
No	83.9	4,155	78.1	4,739		77.7	574	70.2	964		85.9	338	79.7	915	
Yes	16.1	633	21.9	1,144		22.3	177	29.8	421		14.1	65	20.3	243	
**Total**	100.0	4,778	100.0	5,875		100.0	751	100.0	1,385		100.0	403	100.0	1,158	

^a^ Percentages are weighted; Ns are unweighted.

^b^ Bold indicates statistically significant result (*p* < 0.05), italic indicates trending result (*p* < 0.10).

In Nigeria, women with no close confidantes tended to be older than those with one or more confidantes, have less education, and be more likely to be married. Women with no close confidantes also had more children and were less likely to have reported having an abortion (16.1% vs. 21.9%). In Kinshasa, women with no close confidantes were more likely to be married, tended to be less wealthy and have more children, and were less likely to report having had an abortion (22.3% vs. 29.8%). In Kongo Central, women with no confidantes were also less likely to report ever having had an abortion (14.1% vs. 20.3%), though this did not reach statistical significance.

### Known abortion methods

Knowledge of abortion methods was incomplete in all settings, though much higher in Kinshasa and Kongo Central. In Nigeria, respondents reported knowing on average 1.3 methods that women in their community could use to remove an unwanted pregnancy (estimates not shown). However, 42.2% reported knowing no methods (Table 2). In Kinshasa, respondents knew of 2.4 methods on average and only 9.4% reported knowing no abortion methods; women in Kongo Central respondents similarly reported knowing on average 2.3 methods and only 14.2% reported knowing no methods. More than one in four women in Nigeria (27.0%) knew of one or both of the recommended methods for abortion (i.e., medication or procedural abortion), while over half of respondents in the DRC reported knowledge of any recommended methods (Kinshasa: 76.7%; Kongo Central: 58.1%). In all three settings, procedural abortion was much more commonly known than MA. In Nigeria, the most commonly known non-recommended method was ingesting traditional methods such as herbs (24.9%), and in both DRC sites pills used to reduce fever (e.g., antibiotics or antimalarial) were the most well-known non-recommended methods (Kinshasa: 40.0%; Kongo Central: 44.8%).

**Table 2:** Percentage of women ages 15–49 in Nigeria, Kinshasa, and Kongo Central reporting knowledge of specific abortion methods.

	Nigeria		Kinshasa		Kongo Central	
	%^a^	*N*	%	*N*	%	*N*
Method	(*N* = 11,106)		(*N* = 2,136)		(*N* = 1,561)	
Any recommended method	27.0	2,999	76.7	1,671	58.1	943
Procedural	24.7	2,432	72.0	1,527	47.4	816
Medical abortion pills (mifepristone or misoprostol)	9.2	1,113	30.8	669	30.1	409
Pills to reduce fever (antibiotics or antimalarial)	3.6	445	40.0	880	44.8	661
Emergency contraception pills	7.4	779	3.6	68	2.7	36
Other pills	17.2	1,885	25.5	503	31.2	478
Injection	16.1	1,895	29.9	666	26.7	483
Traditional methods, like herbs	24.9	2,988	18.2	460	30.7	466
Ingest industrial product (bleach, Coke-Nescafe)			1.4	29	1.0	15
Alcohol	7.6	672				
Salt, potash, maggi, or kanwa	7.2	902				
Lemon or lime	4.9	702				
Cough syrup	0.2	51				
Insert materials into the vagina	2.2	296	12.3	304	6.2	129
Other	3.7	476	4.6	75	2.0	35
Report no known methods	42.2	4,539	9.4	218	14.2	208

^a^ Percentages are weighted; Ns are unweighted. Percentages do not sum to 100 as multiple responses could be selected. Not all methods were asked about in all study settings.

### Characteristics of women by knowledge of recommended methods

Women with knowledge of recommended abortion methods were statistically significantly different from women without knowledge of recommended methods in all study sites (Table 3). Women who reported knowledge of recommended abortion methods were more likely to report having a close female confidante compared to those who did not know of a recommended method, with the largest difference in Kongo Central (82.2% vs. 67.2%; Kinshasa: 69.1% vs. 64.5%; Nigeria: 61.9% vs. 52.0%). Across all settings, women who knew of recommended abortion methods tended to be older, more educated, and more likely to ever have had an abortion. In Nigeria and Kongo Central, those with recommended method knowledge were less likely to be married than those without such knowledge, while the reverse was true in Kinshasa. In Nigeria and Kongo Central, women who knew of recommended methods tended to have more children than those without such knowledge, while the reverse was true in Kinshasa. Women in Nigeria with knowledge of recommended abortion methods also differed from those without such knowledge in terms of their religion, wealth (wealthier), residence (more likely to live in an urban area), state, and ethnicity.

**Table 3:** Characteristics of women ages 15–49 in Nigeria, Kinshasa, and Kongo Central by whether they reported knowledge of any safe abortion methods (medication, surgical/procedural).

Background characteristics	Nigeria (*N* = 11,106)					Kinshasa (*N* = 2,136)					Kongo Central (*N* = 1,561)				
	Method knowledge		No method knowledge		*p*-value^b^	Method knowledge		No method knowledge		*p*-value	Method knowledge		No method knowledge		*p*-value
	%^a^	*N*	%	*N*		%	*N*	%	*N*		%	*N*	%	*N*	
**Number of close female friends**					**<0**.**001**					**<0**.**001**					**0**.**024**
None	38.1	1,080	48.0	3,708		30.9	552	35.5	347		17.8	198	32.8	205	
One or more	61.9	1,854	52.0	4,029		69.1	1,119	64.5	308		82.2	728	67.2	430	
**Age**					**<0**.**001**					**<0**.**001**					*0*.*061*
15–19	13.3	457	21.2	1,800		15.2	238	44.4	226		17.7	163	24.3	185	
20–24	16.4	517	16.1	1,353		20.8	341	22.5	114		17.1	161	17.1	109	
25–29	21.5	617	17.7	1,423		18.7	306	10.6	54		17.3	170	13.5	78	
30–34	16.6	481	14.4	1,148		13.1	227	7.8	42		14.4	128	12.4	70	
35–39	14.2	417	13.7	1,056		13.4	218	6.6	30		15.8	153	13.5	75	
40–44	11.0	311	10.2	791		11.4	175	5.9	25		9.7	86	10.1	58	
45–49	7.2	199	6.7	536		7.3	125	2.3	15		8.0	65	9.2	60	
**Education**					**<0**.**001**					**0**.**006**					**<0**.**001**
Never	6.5	267	22.0	2,088		0.3	7	0.2	2		3.4	33	10.0	83	
Primary	12.1	420	16.5	1,486		5.7	104	4.5	25		22.7	171	33.5	287	
Secondary	51.4	1,525	45.1	3,409		69.7	1,145	80.4	406		69.0	676	54.8	524	
Higher	30.1	787	16.4	1,124		24.3	374	14.9	73		4.9	63	1.7	19	
**Marital Status**					**0**.**016**					**<0**.**001**					**0**.**037**
Currently married/cohabiting	60.9	1,876	65.4	5,542		45.6	753	27.5	139		55.6	508	63.5	374	
Divorced or separated/widowed	5.2	156	4.1	307		8.1	146	3.8	22		13.9	127	9.0	55	
Never married	33.9	967	30.5	2,258		46.4	731	68.8	345		30.5	291	27.5	206	
**Religion (household)**					**<0**.**001**					0.106					0.171
Catholic	15.1	422	14.5	1,171		14.8	262	18.6	94		25.0	223	18.4	123	
Other Christian	55.2	1,314	39.5	2,509		69.3	1,039	63.0	298		44.4	426	45.6	295	
Muslim	28.2	1,210	43.6	4,159		1.6	22	0.6	2		0.7	8	0.1	1	
Other	1.5	53	2.4	268		14.4	257	17.8	94		29.9	264	35.9	211	
**Wealth**					**<0**.**001**					0.854					**<0**.**001**
Poorest	11.3	419	28.1	2,728		19.0	355	17.0	95		13.4	70	16.1	84	
Second poorest	16.3	563	21.7	1,963		17.7	305	19.1	109		11.8	82	20.4	113	
Middle	19.5	623	16.9	1,304		20.0	324	18.2	95		15.6	122	26.6	149	
Second wealthiest	23.2	651	16.7	1,111		19.6	341	20.4	106		25.3	242	23.0	164	
Wealthiest	29.7	743	16.7	1,001		23.7	305	25.4	101		33.9	410	13.9	125	
**Residence**					**<0**.**001**										
Rural	28.6	1,053	48.7	4,648											
Urban	71.4	1,946	51.3	3,459											
**State**					**<0**.**001**										
Anambra	12.8	381	12.9	1,038											
Kaduna	7.7	610	10.2	2,156											
Kano	8.3	385	15.1	1,366											
Lagos	26.5	560	19.3	1,030											
Nasarawa	9.2	301	15.2	1,235											
Rivers	28.7	615	12.3	608											
Taraba	6.9	147	15.1	674											
**Ethnicity (Nigeria, DRC)**					**0**.**006**					0.605					*0*.*087*
Hausa Bakongo	14.5	760	23.6	2,764		26.2	424	29.1	146		93.8	839	96.9	598	
Igbo Bas-Kasai	25.3	615	21.4	1,456		37.1	644	32.3	175		2.5	35	0.6	11	
Yoruba Kasai	16.1	356	11.9	659		14.9	215	16.9	77		1.2	17	0.9	8	
Other	44.2	1,267	43.1	3,228		21.9	347	21.7	108		2.5	34	1.6	17	
**Parity**					**<0**.**001**					**<0**.**001**					**0**.**002**
0	36.2	1,055	34.6	2,690		37.0	575	63.7	323		22.7	229	24.9	190	
1–2	27.7	784	24.1	1,882		30.8	511	16.1	82		33.4	316	23.6	148	
3–4	22.2	653	21.5	1,732		19.0	314	11.9	64		21.8	195	20.6	119	
5+	14.0	502	19.8	1,788		13.1	229	8.4	37		22.2	185	30.8	178	
**History of abortion**					**<0**.**001**					**<0**.**001**					**<0**.**001**
No	68.1	2,141	86.3	7,155		67.0	1,079	91.0	459		75.5	693	89.1	560	
Yes	31.9	858	13.7	952		33.0	551	9.0	47		24.5	233	10.9	75	
**Total**	100.0	2,999	100.0	8,107		100.0	1,671	100.0	655		100.0	943	100.0	913	

^a^ Percentages are weighted; Ns are unweighted.

^b^ Bold indicates statistically significant result (*p* < 0.05), italic indicates trending result (*p* < 0.10).

Women also differed systematically along several characteristics based on their knowledge of MA pills (Table 4). In all sites, those who knew about MA pills were more likely to have a confidante, though this did not reach significance in Nigeria (Nigeria: 60.8% vs. 54.3%; Kinshasa: 73.6% vs. 65.6%; Kongo Central: 89.0% vs. 70.3%). In all sites, those with MA pills knowledge were significantly more likely to know about procedural abortion, and to report ever having had an abortion in the past. In Nigeria, those with MA pills knowledge tended to be younger and more educated. In Kinshasa, those who knew of MA pills tended to be older, more likely to be married, and more likely to have children, while in Kongo Central, women with MA knowledge were more likely to be Catholic and tended to have fewer children than those who did not know about MA pills.

**Table 4:** Characteristics of women ages 15–49 in Nigeria, Kinshasa, and Kongo Central by whether they reported knowledge of medication abortion methods (using mifepristone and/or misoprostol).

Background characteristics	Nigeria (*N* = 11,106)					Kinshasa (*N* = 2,136)					Kongo Central (*N* = 1,561)				
	MA knowledge		No MA knowledge			MA knowledge		No MA knowledge			MA knowledge		No MA knowledge		
	%^a^	*N*	%	*N*	*p*-value^b^	%	*N*	%	*N*	*p*-value	%	*N*	%	*N*	*p*-value
**Number of close female friends**					*0*.*069*					**0**.**011**					**0**.**019**
None	39.2	410	45.7	4,378		26.4	193	34.5	558		11.0	57	29.7	346	
One or more	60.8	669	54.3	5,214		73.6	476	65.6	909		89.0	352	70.3	802	
**Knowledge of procedural abortion**					**<0**.**001**					**<0**.**001**					**0**.**006**
No	47.0	567	78.2	8,107		15.3	103	33.7	506		35.6	110	59.9	635	
Yes	53.0	546	21.8	1,886		84.8	566	66.3	961		64.4	299	40.1	517	
**Age**					**<0**.**001**					**<0**.**001**					0.124
15–19	12.5	163	19.5	2,094		10.7	67	27.1	397		17.0	64	22.0	284	
20–24	20.2	231	15.8	1,639		21.1	139	21.3	316		16.6	68	17.3	202	
25–29	24.9	261	18.2	1,779		20.6	138	15.1	222		20.3	85	13.7	163	
30–34	16.5	171	14.9	1,458		15.5	114	10.3	155		13.3	58	13.7	140	
35–39	12.5	146	14.0	1,327		15.2	103	10.4	145		16.0	67	14.3	161	
40–44	8.7	90	10.6	1,012		11.5	69	9.5	131		8.5	36	10.4	108	
45–49	4.7	51	7.0	684		5.5	39	6.4	101		8.4	31	8.5	94	
**Education**					**<0**.**001**					0.473					0.193
Never	8.9	112	18.4	2,243		0.1	1	0.3	8		2.4	15	6.6	66	
Primary	14.2	178	15.3	1,728		6.4	48	5.0	81		26.2	76	27.8	290	
Secondary	48.5	537	46.8	4,397		70.4	469	73.0	1,082		65.2	280	63.1	761	
Higher	28.5	286	19.5	1,625		23.1	151	21.7	296		6.2	38	2.5	35	
**Marital Status**					0.618					**0**.**007**					0.573
Currently married/cohabiting	62.6	716	64.3	6,702		47.7	325	38.5	567		57.2	230	59.7	652	
Divorced or separated/widowed	5.1	57	4.4	406		8.0	61	6.6	107		13.7	56	11.0	126	
Never married	32.3	340	31.4	2,885		44.3	283	54.9	793		29.1	123	29.3	374	
**Religion (household)**					0.874					0.273					**0**.**017**
Catholic	14.9	138	14.7	1,455		15.6	113	15.7	243		28.3	120	19.6	226	
Other Christian	46.5	386	43.8	3,437		65.0	405	69.1	932		35.8	154	48.8	567	
Muslim	36.7	563	39.4	4,806		2.2	10	0.9	14		0.2	2	0.6	7	
Other	2.0	26	2.2	295		17.2	122	14.3	229		35.7	133	31.0	342	
**Wealth**					0.111					*0*.*091*					0.503
Poorest	14.5	165	24.1	2,982		23.1	175	16.5	275		18.4	37	12.9	117	
Second Poorest	21.2	251	20.1	2,275		19.1	132	17.5	282		15.3	44	15.5	151	
Middle	21.9	260	17.2	1,667		21.7	141	18.7	278		15.0	52	22.5	219	
Second Wealthiest	28.9	194	18.5	1,568		17.8	126	20.7	321		25.6	116	23.8	290	
Wealthiest	23.5	243	20.2	1,501		18.3	95	26.6	311		25.8	160	25.4	375	
**Residence**					0.120										
Rural	34.1	399	43.8	5,302											
Urban	65.9	714	56.2	4,691											
**State**					0.198										
Anambra	11.1	102	13.0	1,317											
Kaduna	14.8	386	8.9	2,380											
Kano	14.0	175	13.0	1,576											
Lagos	12.8	83	22.3	1,507											
Nasarawa	12.5	111	13.5	1,425											
Rivers	25.3	199	16.2	1,024											
Taraba	9.5	57	13.0	764											
**Ethnicity (Nigeria, DRC)**					0.111					0.830					0.306
Hausa, Bakongo	25.9	430	20.5	3,094		27.0	185	26.8	385		94.0	369	95.6	1,068	
Igbo, Bas-Kasai	20.8	164	22.7	1,907		38.0	276	35.1	543		2.9	18	1.2	28	
Yoruba, Kasai	7.2	66	13.7	949		13.7	71	16.1	221		1.2	7	1.1	18	
Other	46.1	453	43.1	4,042		21.3	137	22.0	318		1.8	15	2.2	36	
**Parity**					*0*.*068*					**<0**.**001**					**0**.**016**
0	36.1	393	35.0	3,352		31.8	199	48.3	699		21.1	94	24.8	325	
1–2	27.9	290	24.8	2,376		36.2	246	23.5	347		34.1	153	27.2	311	
3–4	23.1	247	21.6	2,138		19.5	133	16.4	245		23.9	87	20.2	227	
5+	12.9	183	18.6	2,107		12.5	90	11.8	176		21.0	74	27.9	289	
**History of abortion**					**<0**.**001**					**<0**.**001**					**0**.**005**
No	71.4	822	82.0	8,474		55.4	360	80.3	1,178		71.1	280	85.5	973	
Yes	28.6	291	18.0	1,519		44.6	309	19.7	289		28.9	129	14.5	179	
**Total**		1,113		9,993			669		1,467			409		1,152	

^a^ Percentages are weighted; Ns are unweighted.

^b^ Bold indicates statistically significant result (*p* < 0.05), italic indicates trending result (*p* < 0.10).

### Relationship between having confidantes and knowledge of recommended abortion methods

**Figure 1:**
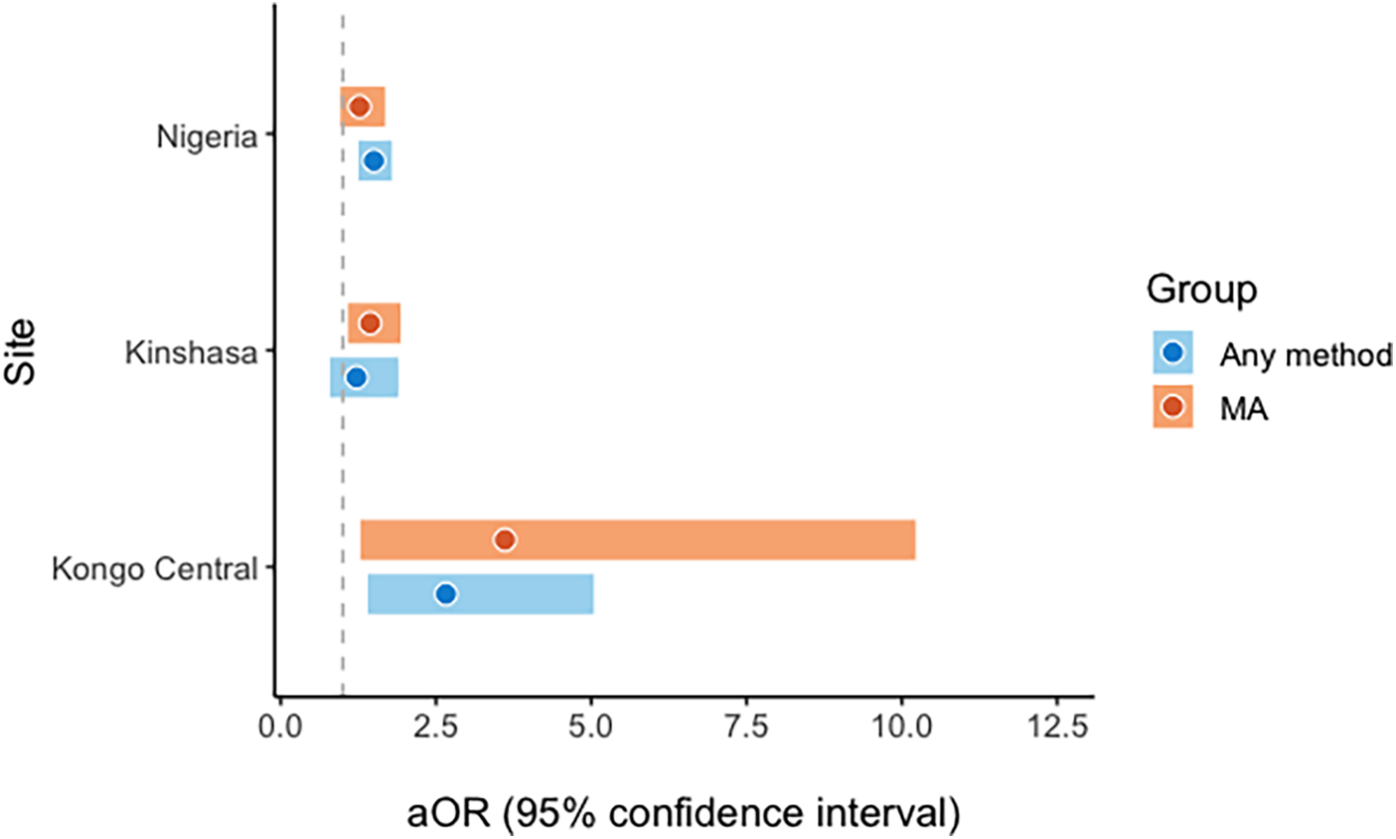
Recommended method knowledge by having a close friend (ref = none).

Multivariate results suggest that having at least one close female confidante is independently associated with significantly increased odds of knowing a recommended abortion method in Nigeria (adjusted odds ratio (aOR) = 1.50, 95% confidence interval (CI) 1.25–1.79) and in Kongo Central (aOR = 2.66, 95% CI 1.40–5.04), but not in Kinshasa (aOR = 1.22, 95% CI 0.79–1.89; Figure 1).

Having close friends was not significantly associated with odds of MA pill knowledge in Nigeria (aOR = 1.27, 95% CI 0.96–1.68), however, it was significantly associated with increased odds of MA pill knowledge in Kinshasa (aOR = 1.44, 95% CI 1.08–1.93) and Kongo Central (aOR = 3.61, 95% CI 1.28–10.22), where the relationship was particularly strong. Sensitivity analyses comparing respondents who knew of MA pills but not procedural abortion to respondents with no knowledge of any recommended methods finds substantial increase in odds of MA pills knowledge associated with having a confidante in Kongo Central (aOR = 8.41, 95% CI 2.32–30.40), which is not replicated in Kinshasa (aOR = 1.34, 95% CI 0.77–2.31); these findings are summarized in the figure shown in Supplementary Figure S2.

## Discussion

The results of this study provide important insight into Nigerian and Congolese women's knowledge of recommended abortion methods and the specific patterns of medication abortion knowledge. While few women in Nigeria know of recommended abortion methods (with even less knowledge of MA), our findings reveal that having a close friend is associated with greater knowledge. The much lower prevalence of MA knowledge may in part be due to the relative recency of including MA in clinical guidelines (and the lack of coordination between private sector supply chains and public sector clinical training), though mifepristone was approved for purposes other than induced abortion much earlier (34).

In the DRC, where recommended method knowledge was much higher, still only one in three respondents knew of MA pills. A recent qualitative study among people who had abortions in Kinshasa indicated that they consulted few people while seeking care to minimize the possibility of being publicly exposed. While some had learned about their options from their own education or medical training, others had learned from their partner, close family members and friends, or women in the community that they knew or suspected had previously had an abortion, indicating that confidantes are just one of several important sources of abortion information used by women in Kinshasa which may explain the lack of significant abortion method knowledge associated with having a confidante in Kinshasa (38).

Together, these findings suggest important variation in the relationship between close friendships and knowledge about safe abortion. Overall knowledge of MA in Nigeria may be too low to penetrate friend networks across existing social strata (e.g., education and ethnicity), such that having close friendships is not likely to offer otherwise unavailable access to MA information. By contrast, three in four women in Kinshasa knew of procedural abortion methods and recommended methods overall, potentially indicating sufficiently widespread knowledge such that informal sharing within close friendships is not instrumental; the lower prevalence of MA knowledge thus aligns with a more significant role of having close friends in Kinshasa. Overall knowledge of recommended abortion methods in Kongo Central falls between Nigeria and Kinshasa, perhaps related to the lower provider density available to rural residents in Kongo Central province compared to Kinshasa, among other social and cultural factors shaping discussion about abortion. Research examining the role of environmental factors in shaping the extent to which individuals discuss their own abortion experiences with social networks suggests that greater abortion visibility can result from a combination of lower abortion stigma and limited ability to anonymously access care (29); efforts to reduce harm related to unsafe abortion in the DRC and the relative scarcity of providers in mixed urban and rural Kongo Central may explain the more significant role of confidantes.

Kongo Central also has the largest group of respondents that reported knowing about MA pills but *not* procedural abortion, and our sensitivity test indicated greatly increased odds of having only MA pill knowledge among those with confidantes in Kongo Central, but not Kinshasa. Importantly, this indicates that information sharing about procedural abortion and MA may be driven by different communication channels in Kongo Central. This again may relate to the varying availability of facilities by site and potentially corresponding prevalence of individuals who have made use of each method. Studies evaluating facility readiness to provide safe abortion care in the DRC find that only 26% of rural facilities are ready to provide this service compared to 50% in urban facilities (35), and that while access to these facilities is common in Kinshasa, it varies by education and wealth in Kongo Central (36). Overall, these findings are consistent with our hypothesis that women who are more socially connected would be more likely to have conversations about sensitive and stigmatizing topics, like abortion, increasing the likelihood they will become aware of recommended abortion methods.

Women in Kongo Central were the most socially connected, with three in four respondents reporting having a close confidante and had the fewest detectable differences between those with and without confidantes; in fact, knowledge of medication abortion was the only statistically significant difference between these groups in Kongo Central. By contrast, there were many factors that differentiated women with close confidante(s) from those without in Nigeria and in Kinshasa. Women with no confidantes were more likely to be married in both sites, which aligns with the challenges in maintaining friendships that arise with competing priorities (encouraged by norms promoting intensive devotion to one's marriage) and potentially relocating to a new community after marriage (37). Respondents with confidantes were older and less educated in Nigeria, and were poorer and more likely to have children in Kinshasa. These patterns suggest that social isolation coincides with other forms of disadvantage that shape access to safe abortion care (35, 36).

Patterns of recommended method knowledge, and of MA pills specifically, differed by study site. Characteristics associated with lower recommended method knowledge in Nigeria align with recent findings indicating subgroups most likely to undergo unsafe abortion (5), suggesting that knowledge may be linked to behavior in this regard. By contrast, women who knew of MA pills specifically did not differ along many characteristics from those who did not know about MA pills; they tended to be younger (excepting the youngest age group), more educated, and also knowledgeable of procedural abortion. Knowledge of MA—which is generally uncommon in this setting—appears to be circulating among slightly different portions of the population than other recommended methods.

In our multivariable analyses in Kinshasa, we find that having a confidante is only significantly associated with MA pill knowledge and not with knowledge of recommended methods in general. This may in part reflect the difference in methods offered by different providers (which may only be a relevant distinction in a context like Kinshasa where there is a sufficient density of known providers of recommended methods): procedural abortion tends to be available at hospitals and clinics, while MA is more commonly available at pharmacies and used by people self-managing their abortion, rendering it less visible (6).

This study has several strengths and limitations. A major strength is that the data come from large, population-based studies that are representative of reproductive-aged women in Nigeria, Kinshasa, and Kongo Central. The data are also rich and allow for adjustment of many potential sociodemographic and reproductive confounders, and include women regardless of their personal abortion history, in contrast to most existing literature. The main limitation is that the question we used to determine knowledge of recommended abortion methods is framed in terms of methods other women in the area use; this does not necessarily reflect the individual's personal knowledge of all abortion methods. For the DRC samples, respondents who did not consent to the abortion survey module were excluded, likely resulting in samples that underrepresent women who are not comfortable talking about abortion. As a result, our findings may exaggerate the role of the confidante because our sample is biased towards those who are open to such conversations or have experienced such conversations with friends. Lastly, we were concerned about the possibility of reverse causality whereby women who have had an abortion are more likely to have become aware of safer abortion methods in the process of seeking care *and* be more likely to have discussed their experience with a friend. However, our sensitivity analysis excluding women who reported having an abortion found no impact on our findings.

This study's findings have implications for programmatic intervention. Results revealed that knowledge of MA pills lags behind knowledge of procedural abortion. MA pills present an opportunity for improved abortion safety outcomes in legally restrictive countries such as Nigeria (15), and those where efforts to expand safe abortion care access are escalating, such as the DRC. Harm reduction efforts that seek to expand knowledge of and access to these medicines can be guided by our findings regarding the specific populations in each setting that are least likely to know about MA, which does not always align with overall knowledge of recommended abortion methods. Our findings regarding the potential role of confidantes in sharing information about abortion methods suggest that programs could spread information more efficiently through social networks, perhaps contingent on community prevalence of method knowledge. Community interventions aimed at reducing abortion stigma and improving knowledge of abortion legality in various settings have recognized the value of empowering community members to carry forward this new information via interpersonal communication in social and clinical settings (39, 40). However, our findings also warn of the potential for socially isolated individuals to be left behind by such programs (particularly populations in humanitarian settings where self-managed abortion and social isolation are both common) (41).

The incomplete and unequal distribution of recommended abortion method knowledge observed in our study reinforces the fact that legalization of abortion alone will not eliminate unsafe abortion. People continue to rely on clandestine providers and self-managed abortion using non-recommended methods in contexts where abortion is broadly legal, unduly putting them at risk for preventable unsafe abortion-related morbidity and mortality (42–44). Conversely, knowledge of recommended abortion methods alone cannot ensure reduced risk of unsafe abortion. Beyond the method, the source (including the training of the provider), the quality of the pills, or the correct use of the pills may all impact actual abortion safety and likelihood of complications. Additionally, knowledge does not guarantee accessibility and affordability. Further research is needed to link this knowledge to subsequent abortion care seeking and outcomes, and to examine the role of social connectedness in sharing information about providers and non-recommended abortion methods.

## Conclusion

The findings in this paper illustrate that Nigerian women generally have low knowledge of recommended abortion methods, and that women in two Congolese provinces have higher but still insufficient knowledge of recommended methods. However, more socially isolated women are even less likely to know about recommended, safer methods for abortion, with distinct patterns shaping knowledge of MA pills in particular. These methods would be least likely to result in abortion-related morbidity and mortality, reducing a woman's risk of experiencing complications if she needs to terminate a pregnancy, and MA is potentially better suited to addressing existing inequities in safe abortion care coverage. Women who were less likely to know of recommended abortion methods share key demographics with women who were more likely to receive an unsafe abortion, namely they tended to be young, poor, and have little to no education. Using a harm reduction framework to improve knowledge of recommended abortion methods, particularly among more socially isolated women, has the potential to reduce unsafe abortion-related negative sequelae.

## Data Availability

The datasets supporting the conclusions of this article are available in the Performance Monitoring for Action repository, https://www.pmadata.org/data/request-access-datasets.

## Footnotes

^1^Questions used in this study come from the female questionnaire abortion modules, which primarily seek to advance methods for measuring abortion incidence and safety using indirect methods. Consequently, surveys feature slight variations in specific survey items. To determine whether using a binary variable instead of a continuous variable to represent respondents’ confidantes in Nigeria, we examined predicted probability of safe abortion knowledge by number of confidantes (see Supplementary Figure S3).

^2^Methods in the “select all that apply” list of methods in the female questionnaire varied by study country, based on prior knowledge and pilot interviews, and full questionnaire text can be found via the PMA website. Methods listed in Nigeria include: surgical procedure, pills called mifepristone or misoprostol (with common brand names), pills used when one has a fever (e.g., antibiotics or antimalarial medicine), emergency contraception (including brand names), pills that do not match any of these categories, injection, traditional methods (like herbs), and several other types of non-recommended methods. In the DRC, this list includes the following categories (as well as several other types of non-recommended methods): surgical procedures, misoprostol alone, misoprostol in combination with mifepristone, emergency contraception, contraceptive pills, medicines you take when you have a fever, other pills, injection, traditional methods that are not inserted into the vagina, inserting materials into the vagina.

^3^We assessed the possibility of variance inflation due to multicollinearity in our model with a VIF test. Some covariates (e.g. state and ethnicity, in Nigeria) had elevated VIFs, but do not impact the association of interest in this study (presence of a close friend and knowledge of safe abortion methods).
