# Supplementary figures and images for: Close female friendships and knowledge of recommended abortion methods in Nigeria and the Democratic Republic of the Congo among a representative sample of reproductive-aged women
Source: Front Reprod Health. 2024 Oct 31;6:1453717. doi: 10.3389/frph.2024.1453717 (PMC11560890; doi:10.3389/frph.2024.1453717)

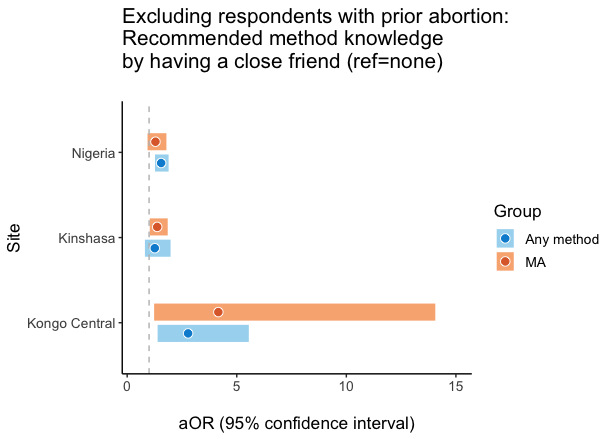

Supplement: Supplementary file 2 [file Supplementaryfile1.png]

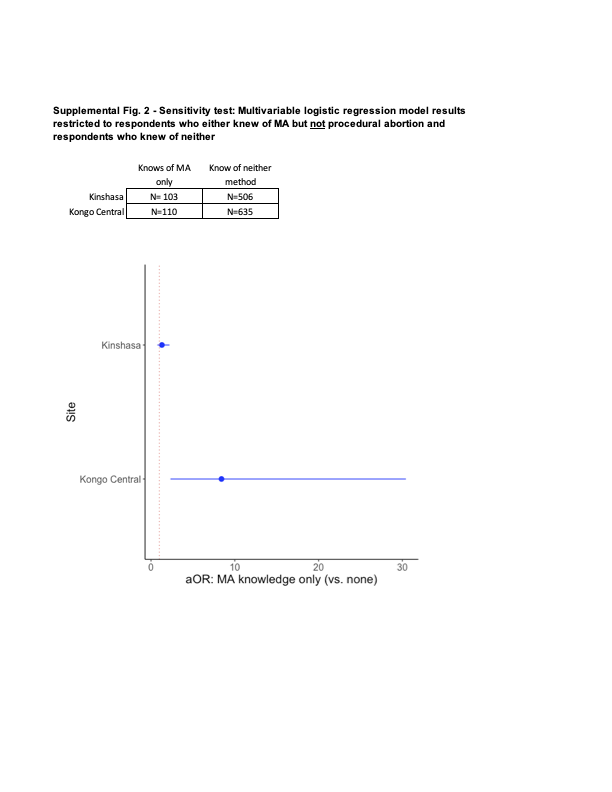

Supplement: Supplementary file 3 [file Supplementaryfile2.png]

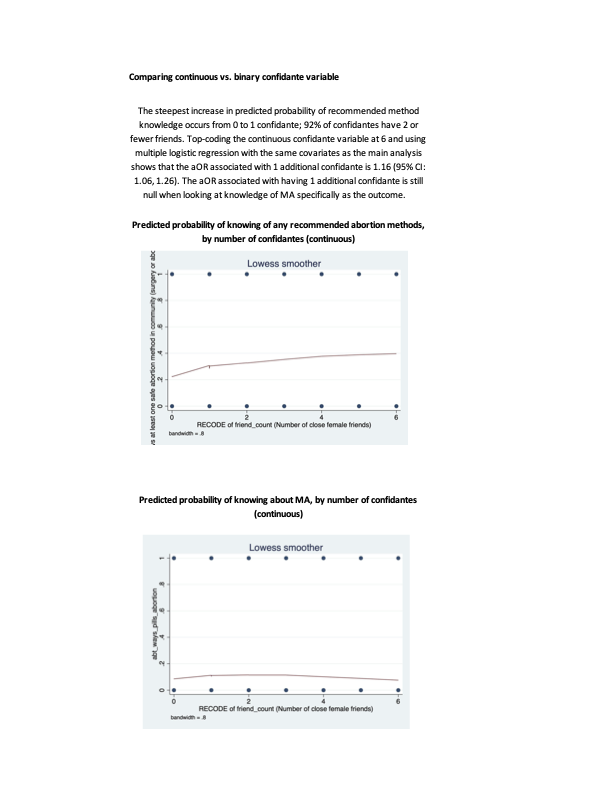

Supplement: Supplementary file 4 [file Supplementaryfile3.png]
